# Job satisfaction and turnover intentions among health care staff providing services for prevention of mother-to-child transmission of HIV in Dar es Salaam, Tanzania

**DOI:** 10.1186/s12960-017-0235-y

**Published:** 2017-09-06

**Authors:** Helga Naburi, Phares Mujinja, Charles Kilewo, Nicola Orsini, Till Bärnighausen, Karim Manji, Gunnel Biberfeld, David Sando, Pascal Geldsetzer, Guerino Chalamila, Anna Mia Ekström

**Affiliations:** 1 Department of Public Health Sciences, Karolinska Institutet, Stockholm, Sweden; 2 Department of Pediatrics and Child Health, Muhimbili University of Health and Allied Sciences (MUHAS), Dar es Salaam, Tanzania; 3 School of Public Health, Muhimbili University of Health and Allied Sciences (MUHAS), Dar es Salaam, Tanzania; 4 Departments of Obstetrics and Gynaecology, Muhimbili University of Health and Allied Sciences (MUHAS), Dar es Salaam, Tanzania; 5 Department of Global Health and Population, Harvard T.H. Chan School of Public Health, Boston, MA United States of America; 6 Africa Health Research Institute (AHRI), Mtubatuba, South Africa; 7 Institute of Public Health, University of Heidelberg, Heidelberg, Germany; 8 Management and Development for Health (MDH) Organisation, Dar es Salaam, Tanzania; 9 Department of Infectious Diseases, Karolinska University Hospital, Huddinge, Stockholm Sweden

## Abstract

**Background:**

Option B+ for the prevention of mother-to-child transmission (PMTCT) of HIV (i.e., lifelong antiretroviral treatment for all pregnant and breastfeeding mothers living with HIV) was initiated in Tanzania in 2013. While there is evidence that this policy has benefits for the health of the mother and the child, Option B+ may also increase the workload for health care providers in resource-constrained settings, possibly leading to job dissatisfaction and unwanted workforce turnover.

**Methods:**

From March to April 2014, a questionnaire asking about job satisfaction and turnover intentions was administered to all nurses at 36 public-sector health facilities offering antenatal and PMTCT services in Dar es Salaam, Tanzania. Multivariable logistic regression models were used to identify factors associated with job dissatisfaction and intention to quit one’s job.

**Results:**

Slightly over half (54%, 114/213) of the providers were dissatisfied with their current job, and 35% (74/213) intended to leave their job. Most of the providers were dissatisfied with low salaries and high workload, but satisfied with workplace harmony and being able to follow their moral values. The odds of reporting to be globally dissatisfied with one’s job were high if the provider was dissatisfied with salary (adjusted odds ratio (aOR) 5.6, 95% CI 1.2–26.8), availability of protective gear (aOR 4.0, 95% CI 1.5–10.6), job description (aOR 4.3, 95% CI 1.2–14.7), and working hours (aOR 3.2, 95% CI 1.3–7.6). Perceiving clients to prefer PMTCT Option B+ reduced job dissatisfaction (aOR 0.2, 95% CI 0.1–0.8). The following factors were associated with providers’ intention to leave their current job: job stability dissatisfaction (aOR 3.7, 95% CI 1.3–10.5), not being recognized by one’s superior (aOR 3.6, 95% CI 1.7–7.6), and poor feedback on the overall unit performance (aOR 2.7, 95% CI 1.3–5.8).

**Conclusion:**

Job dissatisfaction and turnover intentions are comparatively high among nurses in Dar es Salaam’s public-sector maternal care facilities. Providing reasonable salaries and working hours, clearer job descriptions, appropriate safety measures, job stability, and improved supervision and feedback will be key to retaining satisfied PMTCT providers and thus to sustain successful implementation of Option B+ in Tanzania.

**Electronic supplementary material:**

The online version of this article (10.1186/s12960-017-0235-y) contains supplementary material, which is available to authorized users.

## Background

The number of people living with HIV in Tanzania is currently estimated to be 1 400 000 or 4.7 in every 100 people. About 56% of these are women aged 15 years and older [1]. Tanzania is among the 22 Global Plan priority countries identified by the Inter-Agency Task Team (IATT) on prevention and treatment of HIV infection in pregnant women, mothers and children based on the high number (86000) of pregnant women living with HIV who give birth every year [2]. Six years after the launch of the Global Plan (2009–2015), the proportion of pregnant women living with HIV who received antiretroviral medicines doubled, and a threefold decline in the rate of mother-to-child transmission (MTCT) was observed [2]. Implementation of a new strategy, Option B+, thought to be more effective in preventing both MTCT and partner transmission, and not the least for improving maternal health beyond the breastfeeding period, probably contributed to these global achievements [3–5]. The Option B+ strategy requires all HIV-positive pregnant and lactating mothers to begin triple antiretroviral therapy (ART) for life regardless of CD4 cell count or disease stage [3]. In the previous prevention of MTCT (PMTCT) strategies, only pregnant women living with HIV with low CD4 count (< 350 cells/μL) or advanced disease stage (WHO stages III and IV) were offered lifelong ART, while those with a high CD4 count (≥ 350 cells/μL) were only offered antiretroviral prophylaxis until the end of the breastfeeding period [6].

Since approximately 40–50% of all HIV-infected pregnant women have CD4 counts ≤ 350 cells/μL [7, 8], Option B+ will almost double the number of women initiating ART for life in PMTCT clinics [9] and multiply the number of mother-infant pairs who require long-term follow-up as compared to previous PMTCT options. Thus, Option B+ has changed PMTCT service delivery model from providing only prophylaxis to providing a full ART care to pregnant and breastfeeding women living with HIV. This adds other responsibilities to PMTCT providers, such as patient’s clinical assessment, laboratory testing and ART prescription and monitoring both during antenatal and postnatal care [3].

For the full benefit of Option B+ to be realized, all pregnant women who test positive for HIV must be initiated on ART, be retained in care, and be supported to adhere to treatment for life [10]. While Option B+ increases the access to ART for more HIV-positive pregnant women, it does require an expansion of the workforce to match the increased workload [9]. The WHO recommends a minimum of 23 health workers for every 10 000 people as a threshold for providing basic health care [11]. In resource-limited countries such as Tanzania, this is very challenging. Even before the implementation of Option B+, there were only 5 skilled health personnel per 10 000 people [11]. Furthermore, in Tanzanian primary health facilities with PMTCT services, the workload is already almost double compared to primary level facilities without such services [12].

Tanzania started to implement Option B+ for PMTCT of HIV in 2013 [13], followed by a 2% drop in MTCT within 2 years of the implementation [2]. Similar to other Global Plan countries, the final MTCT rate (at 18 months) is still twice the 6-week rate, also after Option B+ was scaled up in Tanzania. A possible explanation is that despite high acceptability and initial uptake of Option B+, there are challenges with adherence to ART and retention in care during the postpartum period and beyond [14].

The gaps in achieving adequate adherence and retention in Option B+ care may be exacerbated by high competing demands on the health care system, coupled with existing staff shortages [15]. The understaffing leads to long queues and increased staff workload, which makes it difficult to efficiently counsel the patients and track the defaulters [16]. Higher workload in already overburdened health systems in resource-limited countries have been linked to a negative effect on health care provider job satisfaction [17, 18], especially if the resulting additional tasks and responsibilities are not coupled to incentives [19].

Provider satisfaction is important for PMTCT because satisfied health workers can deliver higher quality services resulting in better health outcomes and higher patient satisfaction [20–23]. Since patient satisfaction is a determinant of adherence and retention in care [23], improving job satisfaction may play an important role for the implementation of Option B+.

Job satisfaction can reduce work-related stress and thereby employees’ intentions to leave their jobs or to shift workplaces, so-called turnover intentions [24–28]. Given the prevailing staff shortage in health facilities, which is compounded by an increasing workload through Option B+, identifying factors that could affect job satisfaction may be crucial for improving staff motivation, performance and retention.

This study assessed the level of job satisfaction and intention to leave the current job among health care providers in public health facilities offering PMTCT/ANC services in Dar es Salaam, Tanzania. This is important because the success of Option B+ will depend on the active participation of motivated health care providers.

## Methods

### Design

A facility-based, quantitative, cross-sectional study was conducted in 36 public health facilities in Dar es Salaam, Tanzania, between March and April 2014. The health facilities were sampled from all public-sector facilities providing PMTCT services in two (Ilala and Kinondoni) of the three districts in Dar es Salaam, where about 70% of the city’s population reside [29]. The two districts represent the smallest (Ilala) and the largest (Kinondoni) of the three districts of Dar es Salaam both in terms of population and the number of health facilities [30]. The reason for choosing public rather than private clinics is that the majority of the population attends public facilities and most of the workforce shortages are reported from public facilities [11].

During the study period, Tanzania was phasing out Option A (antenatal and intra-partum ART prophylaxis and nevirapine for HIV-exposed infants) and adopting Option B+ following the 2013 WHO recommendations [3]. PMTCT services in Tanzania are routinely provided free of charge and in accordance with the national guidelines that are regularly revised to match the WHO guideline updates [3, 13].

### Sampling

Out of 150 health facilities in these two districts (49 in Ilala and 101 in Kinondoni) providing the integrated antenatal care (ANC) and PMTCT services, 92 non-public facilities were excluded. Ten public facilities were further excluded due to lack of permission from authorities to access them. Out of the remaining public facilities, all 18 facilities from Ilala and another 18 matching (on facility level and size in terms of patient visits) facilities from Kinondoni were selected.

A self-administered questionnaire was distributed to all providers who were present in the PMTCT clinic on the day of the interview. About 2% of the providers were excluded because they did not give consent to participate. Finally, the questionnaires were distributed to 250 health care providers who predominantly provide PMTCT services. These included nurse officers and nurse midwives with a college diploma (trained for at least 3 years), as well as public health nurses and nurse assistants with a college certificate (trained for at least 2 years).

### Questionnaires and piloting

We used a structured questionnaire created and validated in terms of content in Mali and Senegal to ensure its suitability for the sub-Saharan African context [31]. The questionnaire had been developed in four stages, where the first included the testing of four dimensions for measuring job satisfaction based on previous work, the Measure of Job Satisfaction [32] and the Job Descriptive Index [33]. The second stage involved a comprehensive literature review and expert consultations leading to the addition of two more dimensions. The third stage involved testing of the new questionnaire in Senegal and Mali in 2008 and 2010, respectively; after that, the questionnaire was modified by adding three more dimensions. The final stage involved the testing and validation of the final questionnaire using a combined psychometric analysis and expert consultations [31].

The questions were organized into the following categories: socio-demographic characteristics, such as age and gender; professional qualifications; overall job satisfaction; and satisfaction with different aspects of the job. The overall job satisfaction was assessed using one specific question, and the satisfaction with different aspects of the job was assessed using nine domains with a total of 42 questions (minimum 3 and maximum 5 questions per domain). The satisfaction questions (Additional file 1) were Likert-type, based on a 5-point scale coded on a scale from 1 (strongly disagree) to 5 (strongly agree). The questions were formulated to ask if the providers agreed that they were satisfied with various aspects of their job, such as remuneration, equipment and work context, workload, duties, harmony in the workplace, on-the-job training, management, moral satisfaction and job stability. We consulted and obtained permissions to use the scale from the corresponding author Fournier Pierre [31]. In collaboration with language experts, the scale was translated from French to English and finally to Kiswahili (local language).

Questions on health care providers’ intentions to leave their current job and their perceptions on various PMTCT options were developed by the research team, e.g. “How frequently do you think about leaving your current job?”, and the responses were marked on a 7-item Likert scale ranging from 1 (never) to 7 (very often). We also asked the providers about which PMTCT Option (A, B or B+) they themselves thought was the best for the mother and her baby, versus which option they thought would be preferred by the patients versus which option they thought would be most feasible for implementation in the Tanzanian public health system.

To ensure quality and reliability, the data collection tool was piloted at different health facility levels (hospital, health centre and dispensary level), before initiating the study, and the inputs were used to modify and improve the questionnaire.

### Data collection procedure

Twenty Tanzanian research assistants with a medical education background were recruited and trained to understand and use the research protocol and data collection tools.

After obtaining a written consent, the self-administered questionnaire was filled in by the providers during work breaks or after a shift was completed. Providers were informed that participation was voluntary and that they could withdraw from the study at any time. Completing the questionnaire took approximately 45 min, and participants were reimbursed with a small amount equivalent to a 1-h salary, 10 000 Tanzanian shillings ($5), after completing the survey.

To ensure confidentiality, providers were informed to not write their names on the questionnaire to avoid any identifying information. We obtained permission to conduct the survey from both the district and health facility authorities in charge. The Research and Ethics Committee of Muhimbili University of Health and Allied Sciences (MUHAS) gave ethical clearance for the study.

### Analysis

Categorical variables were summarized as proportions, and Fisher’s exact test was used to test for association between various factors and job satisfaction. We calculated the Cronbach alpha for each domain, and the coefficients showed that except for the job stability domain where a coefficient 0.69 suggested fair reliability, the domain scores included in the questionnaire (0.77 to 0.92) were very reliable. For the purpose of this analysis, we converted responses from a 5- or a 7-item Likert scale to binary outcomes.

The primary dependent variable for this analysis was “overall satisfaction with the current job”, with five different response alternatives on a 5-item Likert scale. Health care providers who reported to be “neutral”, “dissatisfied” and “very dissatisfied” were categorized as “dissatisfied”, while those who reported to be “very satisfied” or “satisfied” were categorized as “satisfied”.

The secondary dependent variable was “intention to leave the current job”, with seven possible Likert-scale responses: “very often”, “fairly often”, “often”, “occasionally”, “rarely” and “almost never” were considered as having an intention to leave, while those who reported “never” were considered to have no intention to leave their current job.

The explanatory variable covered job satisfaction in nine domains, converting 5-item Likert-scale replies into binary outcomes dividing the responses into “dissatisfied” (including “neutral”/“dissatisfied” and “very dissatisfied”) versus “satisfied” (“very satisfied” and “satisfied”). Other independent variables included provider baseline characteristics such as age, gender, education level, professional cadre and the time in current position. We also assessed the association between the health care provider’s perception towards various PMTCT options and their job satisfaction or intention to leave their current job, which we thought was of special interest during the policy transition period from Options A and B to Option B+ in Tanzania.

The statements in each domain were used to create separate logistic regression models for satisfaction with the current job and for the intention to leave the current job. We selected one variable with the lowest *p* value, provided that the *p* value was less than 0.2, from each model. For both main outcomes (satisfaction with current job or intention to leave the current job), the independent variables that were significantly associated with the dependent variable were used to create the final multiple logistic regression models using a backward elimination method. Lastly, the relationship between respondents’ satisfaction with their current job and their intention to leave this job was assessed in a separate model. All models were adjusted for cluster effect at the facility level taking into consideration that several health care providers were interviewed at the same facility.

We also examined possible multiplicative interactions between predictors in the final multiple logistic regression models by adding their product terms. A formal *p* value for departure from interaction was obtained using a likelihood ratio test comparing the maximized log-likelihoods of the model with and without the product terms. The results are presented as odds ratios (ORs) and their 95% confidence intervals. The data analysis was done using Stata, release 14 (StataCorp LP; College Station, TX).

## Results

The response rate was 87% (*N* = 217 out of 250 distributed questionnaires), but 4 (2%) were incomplete and excluded from the analysis. Among the 213 questionnaires analysed, we found that 54% (*N* = 115) of the providers in these public PMTCT facilities were dissatisfied with their current job and 35% (*N* = 74) had thought of leaving their current job.

### Baseline characteristics of health care providers

Almost all PMTCT providers (96%) were female and two thirds had a college education: 28% (*N* = 58) were nurse officers, 26% (*N* = 55) were nurse midwives, 32% (*N* = 68) were nurse attendants and the remaining 14% (*N* = 29) either worked in home-based care or were public health nurses. The majority of the providers (81%; *N* = 172) worked in low-level facilities (dispensaries) while the remaining 19% worked in higher-level facilities such as health centres or hospitals. Furthermore, 44% (*N* = 85) of the providers had been employed in the same position for 5 years or more while only 6% were new (had less than a year) on the job (Table 1).

**Table 1:** Socio-demographic characteristics of health care providers working in Dar es Salaam, Tanzania

Variables	Total *N*	Percent
Age in years (*N* = 197)		
< 30	35	18
30–40	73	37
> 40	89	45
Sex (*N* = 192)		
Female	184	96
Male	8	4
Education (*N* = 206)		
Primary school	19	9
Secondary school	38	19
College (certificate)	77	37
College (diploma) and above	72	35
Professional title/cadre (*N* = 210)		
Nurse officer	58	28
Nurse midwife	55	26
Nurse attendant/auxiliary	68	32
PHN/HBC nurse	29	14
Duration in current position (*N* = 194)		
< 1 year	12	6
1–5 years	97	50
5–10 years	42	22
> 10 years	43	22
Distance from home to work (*N* = 199)		
< 1 h	46	23
1–2 h	69	35
> 2 h	84	42
Health facility level (*N* = 213)		
Hospitals and health centres	41	19
Dispensary	172	81

Some variables have missing data; thus, the total in each category does not always add up to *N* = 213

*PMTCT* prevention of mother-to-child transmission of HIV, *HBC* home-based care nurse, *PHN* public health nurse

### Health care provider satisfaction in relation to baseline characteristics

There was no statistically significant difference in terms of socio-demographic characteristics among health care providers who were satisfied compared to those who were dissatisfied with their current job, either in terms of age, education level or professional cadre or in terms of duration in the current position (Table 2).

**Table 2:** Characteristics of health care providers in PMTCT clinics comparing those who were satisfied versus dissatisfied with their current job

Variables	Satisfied *N* (row %)	Dissatisfied *N* (row %)	*p* value
Age in years (*N* = 197)			0.64
< 30	14 (40)	21 (60)	
30–40	37 (51)	36 (49)	
> 40	38 (43)	51 (57)	
Sex (*N* = 192)			0.24
Female	85 (46)	99 (54)	
Male	2 (25)	6 (75)	
Duration in current position (*N* = 194)			0.84
< 1 year	5 (42)	7 (58)	
1–5 years	47 (48)	50 (52)	
5–10 years	17 (40)	25 (60)	
> 10 years	22 (51)	21(49)	
Education (*N* = 206)			0.51
Primary school	9 (47)	10 (53)	
Secondary school	22 (58)	16 (42)	
College (certificate)	36 (47)	41 (53)	
College (diploma) and above	29 (40)	43 (60)	
Professional title/cadre (*N* = 210)			0.17
Nurse officer	27 (47)	31 (53)	
Nurse midwife	20 (36)	35 (64)	
Nurse attendant/auxiliary	38 (56)	30 (44)	
PHN/HBC nurse	12 (41)	17 (59)	
Health facility level (*N* = 213)			0.29
Hospitals and health centres	16 (39)	25 (61)	
Dispensary	83 (48)	89 (52)	
Distance from home to work (*N* = 199)			0.53
< 1 h	25 (54)	21 (46)	
1–2 h	33 (48)	36 (52)	
> 2 h	37 (44)	47 (56)	
Option preferred by women^a^ (*N* = 202)			0.03
PMTCT Option B+	84 (50	84 (50)	
Other PMTCT Options	8 (24)	26 (76)	
Feasible PMTCT Option^a^ (*N* = 197)			0.32
PMTCT Option B+	83 (47)	94 (53)	
Other PMTCT Options	7 (35)	13 (65)	
Best for mother and baby^a^(*N* = 200)			0.64
PMTCT Option B+	88 (49)	91 (51)	
Other PMTCT Options	4 (19)	17 (81)	

Some variables have missing data; thus, the total in each category does not always add up to *N* = 213

*PMTCT* prevention of mother-to-child transmission of HIV, *HBC* home-based care nurse, *PHN* public health nurse

^a^Health care workers opinions regarding Option B+

When asked about their views on various PMTCT options, most providers were in favour of Option B+ rather than the short-term PMTCT options that were being phased out (Options A and B). Providers who thought Option B+ rather than the other options was the best alternative for women living with HIV and their babies were less likely to be dissatisfied with their current job (51% compared to 81% were dissatisfied, respectively, *p* = 0.64), and among those who thought that Option B+ rather than the other options would be preferred by the clients themselves (50% compared to 76% were dissatisfied with their job, *p* = 0.03) indicating that providers with high acceptability towards Option B+ also felt better about their work (Table 2).

### Health care provider turnover intentions in relation to baseline characteristics

Not unexpected, intention to leave the current job was high for providers who also were dissatisfied with their current job: 45% (*N* = 66) of those who were dissatisfied with their current job compared to 27% (*N* = 34) of providers who reported to be satisfied in their current position thought of leaving their current job.

The intention to leave the current job was not influenced by age, educational level and the duration in current position, professional cadre or travel distance to work. Providers who worked in the lowest-level facilities, the dispensaries, were less likely to consider leaving their current job compared to those employed in hospitals or health centres (31% compared to 59%, *p* = 0.01). Those who thought that women living with HIV would prefer Option B+ rather than other options (32% compared to 62%, *p* = 0.04) were also less likely to think about leaving their current job **(**Table 3.

**Table 3:** Characteristics of health care providers in PMTCT clinics comparing those who had an intention to leave their current job versus those with no such intention

Variables	Intention to leave current job: *N* (%)	No intention to leave: *N* (%)	*p* value
Age in years (*N* = 191)			0.05
< 30	15 (43)	20 (57)	
30–40	34 (48)	37 (52)	
> 40	20 (24)	65 (76)	
Sex (*N* = 185)			0.41
Female	4 (57)	3 (43)	
Male	60 (34)	118 (66)	
Duration in current position (*N* = 186)			0.71
< 1 year	7 (58)	5 (42)	
1–5 years	35 (38)	56 (62)	
5–10 years	12 (29)	29 (71)	
> 10 years	16 (38)	26 (62)	
Education (*N* = 199)			0.25
Primary school	3 (17)	14 (82)	
Secondary school	7 (18)	31 (82)	
College (certificate)	30 (42)	42 (58)	
College (diploma) and above	33 (46)	39 (54)	
Professional title/cadre (*N* = 202)			0.65
Nurse officer	18 (32)	38 (68)	
Nurse midwife	21 (41)	30 (59)	
Nurse attendant/auxiliary	21 (31)	46 (69)	
PHN/HBC nurse	14 (50)	14 (50)	
Health facility level (*N* = 213)			0.01
Hospitals and health centres	24 (59)	17 (41)	
Dispensary	53 (31)	119 (69)	
Distance from home to work (*N* = 191)			0.14
< 1 h	14 (31)	31 (69)	
1–2 h	28 (42)	39 (58)	
> 2 h	13 (16)	66 (84)	
Option preferred by women^a^ (*N* = 198)			0.04
PMTCT Option B+	52 (32)	112 (68)	
Other PMTCT Options	21 (62)	13 (38)	
Feasible PMTCT Option^a^ (*N* = 194)			0.87
PMTCT Option B+	63 (36)	111 (64)	
Other PMTCT Options	8 (40)	12 (60)	
Best for mother and baby^a^ (*N* = 196)			0.51
PMTCT Option B+	62 (35)	113 (65)	
Other PMTCT Options	10 (48)	11 (52)	

Some variables have missing data; thus, the total in each category does not always add up to *N* = 213

*PMTCT* prevention of mother-to-child transmission of HIV, *HBC* Home Based Care nurse, *PHN* Public Health Nurse

^a^Health care workers opinions regarding Option B+

### Health care workers’ satisfaction with different aspects of their job

Different job aspects were organized into nine different domains, including remuneration, equipment and work context, workload, duties, harmony in the workplace, training and management, moral satisfaction and job stability (being able to keep the same job for a long time).

Among the nine domains of job satisfaction, none or very few of the providers responded “very satisfied” or “satisfied”, to any of the 5 questions on remuneration and workload domains. For the other domains, a few providers said they were “very satisfied” and a fair number responded “satisfied” to most of the questions in the domains covering availability of equipment, job stability and management. Moral satisfaction, type of duties and harmony in workplace stood out as the most satisfying areas since most health workers responded, “very satisfied” or “satisfied” to these domain questions (Additional file 2: Figure S1).

### Factors associated with satisfaction with the current job among PMTCT providers

Domain-specific significant predictors with a *p* value below 0.2 were retained in the final multiple regression model. Provider opinions on women’s preference for Option B+ were included in the final model because these were significantly and inversely associated with workers being dissatisfied with their current job. The final model was adjusted for age, education level, level of health care facility and professional cadre, as well as for clustering of standard errors at the facility level.

The adjusted odds ratios (aORs) in the final model showed that being dissatisfied with the salary (aOR 5.6, 95% CI 1.2–26.8), with the job description (aOR 4.3, 95% CI 1.3–14.7), with the availability of protective gears (aOR 4.0, 95% CI 1.5–10.6) and with working hours (aOR 3.2, 95% CI 1.3–7.6), was significantly associated with also being dissatisfied with the current job.

Providers who thought that HIV-positive women would prefer the PMTCT option they were now promoting, i.e. Option B+, had 80% lower odds of dissatisfaction (aOR 0.2, 95% CI 0.1–0.9) compared to providers who thought that women would prefer other options (Table 4). There was no significant interaction between any of these important predictors (all *p* > 0.05).

**Table 4:** Predictors of overall job dissatisfaction among health care providers working in PMTCT clinics in Dar es Salaam, Tanzania

Variable	Crude odds ratio	Adjusted odds ratio
	(95% CI)	(95% CI)
Dissatisfaction with		
Amount of salary	7.9 (1.3–45.7)	5.6 (1.2–26.8)
Job description	6.5 (3.0–14.0)	4.3 (1.3–14.7)
Availability of protective gear	3.7 (1.7–8.2)	4.0 (1.5–10.6)
Working hours	4.9 (2.5–9.8)	3.2 (1.3–7.6)
New knowledge from work training	3.4 (2.0–5.8)	1.9 (0.8–4.8)
Institutional management information	2.0 (1.1–3.7)	–
Recognition of work by colleagues	2.7 (1.1–3.7)	–
Quality of one’s own work	3.7 (1.2–11.2)	–
Employment status	3.2 (1.52–6.8)	–
Health care workers opinion on Option B+		
Women clients prefer Option B+	0.3 (0.1–0.7)	0.2 (0.1–0.9)
Option B+ is the best for mothers and babies	0.2 (0.6–0.9)	–

Crude odds ratio: univariate associations between each of the explanatory variables and the outcome variable. Adjusted odds ratio: associations between the explanatory variables and the outcome variable found to have a *p* value < 0.2 in the univariate models, adjusted for age, education level, level of health facility and professional cadre in a multivariate model. Empty cells represents results overlapping the null value (OR = 1). All models are adjusted for clustering of standard errors at the health care facility level

### Factors associated with the intention to leave the current job among PMTCT providers

All domain-specific predictors of intention to leave the current job with a *p* value below 0.2 were retained in the final multiple regression model. This model was also adjusted for age, education level, level of health care facility and professional cadre, as well as for clustering of standard errors at the facility level.

In the final multivariate model, we found that those providers who were dissatisfied with the job stability (aOR 3.7, 95% CI 1.3–10.5) or with recognition from the boss (aOR 3.6, 95% CI 1.7–7.6) or were unhappy with the information provided to them on institutional performance (aOR 2.7, 95% CI 1.3–5.8) were also those who had an intention to leave their current job (Table 5). There was no significant interaction between these predictors (all *p* > 0.05).

**Table 5:** Predictors of intention to leave the current job among health care providers who work in PMTCT clinics in Dar es Salaam, Tanzania

Variable	Crude odds ratio OR (95% CI)	Adjusted odds ratio OR (95% CI)
Dissatisfaction with the		
Amount of salary	3.8 (1.1–14.1)	–
Availability of job aids	2.3 (0.9–5.2)	–
Allocation of workload between team members	2.5 (1.1–5.7)	–
Job description in relation to actual work	3.2 (1.7–6.2)	–
Recognition of work by boss	4.4 (2.3–8.4)	3.6 (1.7–7.6)
Knowledge from on-the-job training	2.2 (1.2–4.0)	–
Performance of the department	4.7 (2.5–8.8)	2.7 (1.3 - 5.8)
ANC/PMTCT outcomes	3.9 (1.7–8.8)	–
Job stability	4.1 (2.0–8.3)	3.7 (1.3–10.5)
Health facility level		
Hospital compared to dispensary	3.1 (1.2–8.0)	–
Dispensary	1	1
Health care workers opinion on Option B+		
Women clients prefer Option B+	0.3 (0.1–0.8)	–
Option B+ is the best for mothers and babies	0.7 (0.2–2.0)	–
Option B+ is feasible to implement in Tanzania	0.9 (0.3–3.0)	–

Crude odds ratio: univariate associations between each of the explanatory variables and the outcome variable. Adjusted odds ratio: associations between the explanatory variables and the outcome variable found to have a *p* value < 0.2 in the univariate models, adjusted for age, education level, level of health facility and professional cadre in a multivariate model. Empty cells represents results overlapping the null value (OR = 1). All models are adjusted for clustering of standard errors at the health care facility level

## Discussion

This study reveals that more than half of all PMTCT health care providers interviewed in the study (the majority of them were working with PMTCT Option B+) were dissatisfied with their current job and one third of them thought about leaving their current job. Dissatisfaction with the current job was strongly associated with being dissatisfied with the level of salary, having inadequate protective gear, having an unclear job description and a feeling of unreasonably long working hours. Actually expressing an intention to leave the current job was associated with other factors such as a sense of poor job stability, poor employee recognition and inadequate feedback on the overall performance of the workplace.

Both dissatisfaction with and intention to leave the current job were independent of age, education or seniority/level of training (health worker cadre) among the PMTCT staff interviewed in Dar es Salaam. Thus, although reasons for job satisfaction and intention to leave one’s job may differ between contexts, work-related factors that can be modified such as offering a reasonable salary, access to personal protection equipment and good leadership and management remain more important than any individual characteristics of the providers [34–36].

This means that in order to retain existing staff and to attract new health care providers to work with antenatal care and PMTCT in particular, it is also necessary to invest in management and leadership skills in the health care sector. In addition to ensuring a sense of security through higher job stability, interventions aimed at improving the workplace atmosphere such as higher transparency in terms of decision-making and more positive feedback on an individual provider’s work as well as on the overall performance of the clinic are very important. This is especially important today since Option B+ has changed the PMTCT service delivery model from providing short-term MTCT prophylaxis to full ART care aimed to continue medications for life. Thus, the new PMTCT strategy has not only increased the patient load but also resulted in additional tasks at the antenatal care level such as clinical monitoring of the HIV infection, drawing blood samples (for CD4 and viral load tests) and prescribing ART, tasks that previously were carried out in ART clinics. However, despite such an inevitable increase in workload, Option B+ was the preferred option among the majority of health personnel in our study. Most thought Option B+ had a higher potential for effective prevention of MTCT than previous strategies [4, 37, 38], to improve the long-term health of women and children and eliminate MTCT if implemented efficiently.

To effectively implement Option B+, satisfied and motivated providers is key, since they must be highly committed to spend adequate time with the clients to inform them about the PMTCT program and also to provide individual counselling, emphasizing the importance of adherence to medication (for both mothers and their infants) and retention in ART care [16].

Previous studies from African countries have cited dissatisfaction with remuneration as the most important factor to enhance staff motivation and retention [39–42]. Also in the current study, most providers reported that they were dissatisfied with their salaries in turn increasing their level of overall job dissatisfaction almost sixfold. However, in addition to the remuneration, which is highly resource-constrained, other factors that should be easier to improve such as unclear job descriptions, inadequate availability of the protective materials such as gloves, and long working hours also independently contributed to job dissatisfaction.

The availability of adequate and functional personal protective materials is particularly essential in HIV service delivery where both providers and patients must feel that the providers are adequately protected [43] alleviating stress and their fear of HIV transmission [44, 45].

Furthermore, we found that providers who were dissatisfied with their workload were three times more likely to be dissatisfied with their current job. A heavy workload, most often due to the shortage of health workers (the majority being women), cuts the time that would otherwise be available for family duties and recreation, in turn increasing their risk of stress-related morbidity, absenteeism and burnout [17]. Thus, apart from inadequate salary and supplies [43, 46], a heavy workload [46, 47] and an unfavourable working environment [48, 49] are important reasons for job dissatisfaction and attrition of health personnel.

Our findings add to the current evidence that illustrates the multidimensional nature of job satisfaction and dissatisfaction and the variation between different contexts [50, 51], but it also further underlines the importance of the work environment [46, 47, 52].

Our study did not find a significant impact of inadequate training and supervision on overall job dissatisfaction as reported previously by others [47, 53]. The high rates of salary dissatisfaction could have overshadowed this factor, but perhaps the training and active supervision provided to all PMTCT providers in preparation for the implementation of Option B+ made them feel more ready for the task. The fact that providers who thought that their clients would prefer Option B+ were less likely to be dissatisfied with their current job probably reflects the importance of moral gratification in relation to patient satisfaction. It could also reflect the health providers’ hopes and expectations that Option B+ could result in less effort invested into enhancing adherence and tracking defaulters in the long term, consequences that are yet to be evaluated.

The intention to leave the current job in our study was largely influenced by job instability and the fear of losing one’s job. This finding was unexpected since public-sector employment is permanent and pensionable in Tanzania. We speculate that this could be a reflection of either poor communication between employers and employees and/or the routine practice in Tanzania to rotate staff between different public health facilities. Staff rotation is done with the intention to reduce work-related stress and burnout and improve satisfaction among the providers [54] but can obviously also affect health workers’ motivation especially when it poses regular re-training challenges and constant re-adaption to new work environments [55]. In the present study, other factors for the intention to leave one’s current job include dissatisfaction with the way superiors value or recognize the work one performed and poor feedback regarding the overall performance of the clinic. Health care managers need to be made more aware and educated on the importance of good leadership and management that recognize employees’ work and provide timely feedback on both individual performance and the performance of the unit as a whole in order to encourage workplace retention and to improve health service delivery [56–58].

Thus, we conclude that optimal implementation of Option B+ and desired MTCT outcomes will require more supportive leaders, improved working conditions and staff communication in parallel with increased remuneration.

A possible limitation was the fact that our results may have been influenced by the fairly short work experience providers had of Option B+ since the study was conducted during the transition from Option A to Option B+. Their positive views on Option B+ could also have been biased by recently having received the on-the-job trainings related to Option B+ implementation. This could have influenced their level of satisfaction with trainings and their positive expectations of Option B+; however, it is unlikely to have had any influence on other aspects of job satisfaction. Additionally, the study used a cross-sectional design, which does not allow conclusions about causal relationships. However, we believe that our findings contribute to the existing knowledge on a crucial area for the public sector in Tanzania and similar contexts in East Africa where optimizing the conditions for successful implementation of PMTCT Option B+ is of high importance for millions of women and children.

## Conclusion

We found that although remuneration is crucial for job satisfaction, there are also other important factors that influence both job satisfaction and intention to leave the job.

Thus, in order to recruit and retain satisfied PMTCT providers to sustain Option B+ in Tanzania, deliberate efforts should be directed to making public clinics more attractive workplaces by ensuring reasonable salaries and working hours, clearer job descriptions, appropriate safety measures and job stability as well as ensuring adequate management skills.

## Additional files

Additional file 1: Questionnaire on health care provider job satisfaction. (DOCX 102 kb)

Additional file 2: Health care provider satisfaction by work domain responding to the question “How satisfied are you with…” on a 5-item Likert scale. (DOCX 51 kb)

## Abbreviations

ANC: Antenatal care
aOR: Adjusted odds ratio
ART: Antiretroviral therapy
HIV: Human immunodeficiency virus
HRH: Human resource for health
IATT: Inter-Agency Task Team
MDH: Management and Development for Health
MTCT: Mother-to-child transmission of HIV
MUHAS: Muhimbili University of Health and Allied Sciences
OR: Odds ratio
PMTCT: Prevention of mother-to-child transmission of HIV
Sida: Swedish International Development Cooperation Agency
WHO: World Health Organization
