# Supplementary material for: Job satisfaction and turnover intentions among health care staff providing services for prevention of mother-to-child transmission of HIV in Dar es Salaam, Tanzania
Source: Hum Resour Health. 2017 Sep 6;15:61. doi: 10.1186/s12960-017-0235-y (PMC5585985; doi:10.1186/s12960-017-0235-y)
Supplement: Supplementary file 1 — Questionnaire on health care provider job satisfaction. (DOCX 102 kb) [file 12960_2017_235_MOESM1_ESM.docx]

## **Questionnaire on health care provider job satisfaction:**

Date: ____________________

Name of health facility/ ward: _______________________________________

Code no of investigator: __________________________________________

| *Management and Development for Health (MDH) is currently carrying out the Familia Salama trial. As part of MDH’s evaluation of the trial, we would like to find out about your experience with your work as a nurse, CBHC / HBC. This questionnaire is anonymous and individual questionnaires will only be seen and analyzed by MDH’s data management team. It should take approximately 30 -45 minutes to Complete this form.*  This survey is anonymous and the answers from many clinics will be analysed together as a group, thus it will not be possible to link you to your answers. |
| --- |

**PART 1. SOCIO DEMOGRAPHIC CHARACTERISTICS OF HEALTH CARE WORKERS**

|  | Age | _____years |
| --- | --- | --- |
|  | Sex:   1. Male 2. Female |  |
|  | What is the highest level of formal education you have completed in school?   1. None 2. Primary School 3. Secondary School 4. College (certificate) 5. College (diploma) 6. University |  |
|  | What is your professional title:   1. Nurse officer 2. Nurse midwife 3. Auxiliary nurse 4. Nurse attendant 5. Public health nurse 6. Nurse counselor 7. Nutritionist 8. HBC 9. Other - specify ______________ |  |
|  | How long have you worked in your  *current position* | ___yrs ___months |
|  | *If less than 1 year in your current position, specify whether this is due to:*   1. New recruitment 2. Change of department or institution 3. Promotion |  |
|  | Do you have any other job(s) apart from the work you do here?   1. Yes (specify----------------------------) 2. No |  |
|  | How long does it take to get to the place of work from your house | ____hrs ___min |

| **Instructions: The questions below relate to different job satisfaction. For each question, there are 5 and in other 7 choices of answer, there are no correct or wrong answers; what is important is that you express your opinion** |
| --- |

**PART 2: SATISFACTION WITH WORK.**

|  | **Questions on overall, job satisfaction.** | 5  Very Satisfied | 4  Satisfied | 3 Neutral | 2  Dissatisfied | 1  Very Dissatisfied |
| --- | --- | --- | --- | --- | --- | --- |
|  | Overall, how satisfied are you with your current job? | ☐ | ☐ | ☐ | ☐ | ☐ |

|  | **Questions on remuneration** | 5  Very Satisfied | 4  Satisfied | 3 Neutral | 2  Dissatisfied | 1  Very Dissatisfied |
| --- | --- | --- | --- | --- | --- | --- |
|  | Are you satisfied with the level of your salary? | ☐ | ☐ | ☐ | ☐ | ☐ |
|  | Are you satisfied with allowances (e.g extra duty, outfit, medical & Transport) that you receive? | ☐ | ☐ | ☐ | ☐ | ☐ |
|  | Are you satisfied with the level of coverage of your needs by your salary | ☐ | ☐ | ☐ | ☐ | ☐ |
|  | Are you satisfied with the amount of your salary with regard to your skills? | ☐ | ☐ | ☐ | ☐ | ☐ |
|  | Are you satisfied with the amount of your salary in relation to the volume of your work? | ☐ | ☐ | ☐ | ☐ | ☐ |

|  | **Questions on equipment and work context** | 5  Very Satisfied | 4  Satisfied | 3 Neutral | 2  Dissatisfied | 1  Very Dissatisfied |
| --- | --- | --- | --- | --- | --- | --- |
|  | Are you satisfied with the availability of HIV testing kits? | ☐ | ☐ | ☐ | ☐ | ☐ |
|  | Are you satisfied with the availability of medicines that you need to do your work? | ☐ | ☐ | ☐ | ☐ | ☐ |
|  | Are you satisfied with the availability of consumables (e.g.: cotton, alcohol, etc.) that you need to do your work? | ☐ | ☐ | ☐ | ☐ | ☐ |
|  | Are you satisfied with the availability of protection gear against professional risks (e.g. against exposure to HIV, against other risks)? | ☐ | ☐ | ☐ | ☐ | ☐ |
|  | Are you satisfied with the printed materials available to you for doing your work (job aids including guidelines and chart booklets)? | ☐ | ☐ | ☐ | ☐ | ☐ |

|  | **Questions on workload** | 5  Very Satisfied | 4  Satisfied | 3 Neutral | 2  Dissatisfied | 1  Very Dissatisfied |
| --- | --- | --- | --- | --- | --- | --- |
|  | Are you satisfied with your working hours? | ☐ | ☐ | ☐ | ☐ | ☐ |
|  | Are you satisfied with your workload (quantity of work, overloaded or not)? | ☐ | ☐ | ☐ | ☐ | ☐ |
|  | Are you satisfied with the allocation of the workload between your team members? | ☐ | ☐ | ☐ | ☐ | ☐ |
|  | Are you satisfied with the division of your working hours between various healthcare duties (e.g patient care and report writing)? | ☐ | ☐ | ☐ | ☐ | ☐ |
|  | Are you satisfied with the support provided by the other members of the team for the performance of your duties? | ☐ | ☐ | ☐ | ☐ | ☐ |

|  | **Questions on duties** | 5  Very Satisfied | 4  Satisfied | 3 Neutral | 2  Dissatisfied | 1  Very Dissatisfied |
| --- | --- | --- | --- | --- | --- | --- |
|  | Are you satisfied with the variety of your duties (duties of different kinds)? | ☐ | ☐ | ☐ | ☐ | ☐ |
|  | Are you satisfied with the appropriateness of your duties and your skills (Do your duties correspond well to your skills)? | ☐ | ☐ | ☐ | ☐ | ☐ |
|  | Are you satisfied with the level of professional responsibility entrusted to you? | ☐ | ☐ | ☐ | ☐ | ☐ |
|  | Are you satisfied with your job description? | ☐ | ☐ | ☐ | ☐ | ☐ |
|  | Are you satisfied with what is in your job description and what you actually do? | ☐ | ☐ | ☐ | ☐ | ☐ |

|  | **Questions on harmony in the workplace** | 5  Very Satisfied | 4  Satisfied | 3 Neutral | 2  Dissatisfied | 1  Very Dissatisfied |
| --- | --- | --- | --- | --- | --- | --- |
|  | Are you satisfied with the harmony between the members of your department? | ☐ | ☐ | ☐ | ☐ | ☐ |
|  | Are you satisfied with the way the quality of your work is recognised by your ***colleagues***? | ☐ | ☐ | ☐ | ☐ | ☐ |
|  | Are you satisfied with the recognition of the quality of your work by your superiors/***bosses*** | ☐ | ☐ | ☐ | ☐ | ☐ |
|  | Are you satisfied with the form of grading for promotions? | ☐ | ☐ | ☐ | ☐ | ☐ |
|  | Are you satisfied with the respect with which your superiors/bosses treat you? | ☐ | ☐ | ☐ | ☐ | ☐ |

|  | **Questions on continuous training** | 5  Very Satisfied | 4  Satisfied | 3 Neutral | 2  Dissatisfied | 1  Very Dissatisfied |
| --- | --- | --- | --- | --- | --- | --- |
|  | Are you satisfied with the training that you are ***continuing*** to receive? | ☐ | ☐ | ☐ | ☐ | ☐ |
|  | Are you satisfied with the form of selection of members of your department/team for participation in training activities? | ☐ | ☐ | ☐ | ☐ | ☐ |
|  | Are you satisfied with the appropriateness of the proposed training and your needs? | ☐ | ☐ | ☐ | ☐ | ☐ |
|  | Are you satisfied with the way in which the knowledge acquired in training is used in your work? | ☐ | ☐ | ☐ | ☐ | ☐ |
|  | Are you satisfied with the skills newly acquired during training sessions? | ☐ | ☐ | ☐ | ☐ | ☐ |

|  | **Questions on management** | 5  Very Satisfied | 4  Satisfied | 3 Neutral | 2  Dissatisfied | 1  Very Dissatisfied |
| --- | --- | --- | --- | --- | --- | --- |
|  | Are you satisfied with the positive reinforcement in your workplace? | **☐** | **☐** | **☐** | **☐** | **☐** |
|  | Are you satisfied with the possibilities for participating in decision-making in order to resolve work organisation problems? | **☐** | **☐** | **☐** | **☐** | **☐** |
|  | Are you satisfied with the information given about the performance of your department? | **☐** | **☐** | **☐** | **☐** | **☐** |
|  | Are you satisfied with the information given about your ***institution*** (problems, activities, decisions, financial management, etc.) | **☐** | **☐** | **☐** | **☐** | **☐** |
|  | Are you satisfied with the transparency of management of the financial resources of the institution? | **☐** | **☐** | **☐** | **☐** | **☐** |

|  | **Questions on moral satisfaction** | 5  Very Satisfied | 4  Satisfied | 3  Neutral | 2  Dissatisfied | 1  Very Dissatisfied |
| --- | --- | --- | --- | --- | --- | --- |
|  | Are you satisfied with the outcome of ANC/PMTCT in your department (health of women and new-born babies)? | **☐** | **☐** | **☐** | **☐** | **☐** |
|  | Are you satisfied with the quality of your own work? | **☐** | **☐** | **☐** | **☐** | **☐** |
|  | Are you satisfied with the popular image of your profession? | **☐** | **☐** | **☐** | **☐** | **☐** |

|  | **Questions on stability** | 5  Very Satisfied | 4  Satisfied | 3  Neutral | 2  Dissatisfied | 1  Very Dissatisfied |
| --- | --- | --- | --- | --- | --- | --- |
|  | Are you satisfied with the regularity of payment of your salary (salary paid on time, late, etc.)? | **☐** | **☐** | **☐** | **☐** | **☐** |
|  | Are you satisfied with your job stability (certainty or uncertainty with keeping/losing your job)? | **☐** | **☐** | **☐** | **☐** | **☐** |
|  | Are you satisfied with the regularity of payment of your allowances? | **☐** | **☐** | **☐** | **☐** | **☐** |
|  | Are you satisfied with your type of status (civil servant or contractual terms)? | **☐** | **☐** | **☐** | **☐** | **☐** |

**Additional questions:**

|  | **Intent to leave a job** | Very often | Fairly often | Often | Occasionally | Rarely | Almost never | Never |
| --- | --- | --- | --- | --- | --- | --- | --- | --- |
|  | How frequently do you think about leaving your current job? | ☐ | ☐ | ☐ | ☐ | ☐ | ☐ | ☐ |

1. As a health care provider, which of the three PMTCT options do you think HIV-infected pregnant women will prefer? *(Please circle one.)*

| Option A | 1 |
| --- | --- |
| Option B | 2 |
| Option B+ | 3 |
| I don’t know | 4 |
|  |  |

1. As a health care provider, which of the three PMTCT options do you think will be best for HIV-infected pregnant women and their infants? *(Please circle one.)*

| Option A | 1 |
| --- | --- |
| Option B | 2 |
| Option B+ | 3 |
| I don’t know | 4 |

1. As a health care provider, which of the three PMTCT options do you think is most feasible in the public-sector health system in Tanzania? *(Please circle one.)*

| Option A | 1 |
| --- | --- |
| Option B | 2 |
| Option B+ | 3 |
| I don’t know | 4 |
