# Supplementary material for: Job satisfaction and turnover intentions among health care staff providing services for prevention of mother-to-child transmission of HIV in Dar es Salaam, Tanzania
Source: Hum Resour Health. 2017 Sep 6;15:61. doi: 10.1186/s12960-017-0235-y (PMC5585985; doi:10.1186/s12960-017-0235-y)
Supplement: Supplementary file 2 — Health care provider satisfaction by work domain responding to the question “How satisfied are you with…” on a 5-item Likert scale. (DOCX 51 kb) [file 12960_2017_235_MOESM2_ESM.docx]

**Supplementary figure** **(S1):** Health care provider satisfaction by work domain responding to the question “How satisfied are you with…”) on a 5 item Likert scale
